# Longitudinal changes in MMN and P3 during emotional processing in adolescents who engage in NSSI: a 12-week follow-up study

**DOI:** 10.3389/fpsyt.2026.1871678

**Published:** 2026-06-10

**Authors:** Jianqiang Bi, Pei Liu, Zhenpeng Xue, Xiujuan Li, Qiang Xu, Yanbin Jia, Jianping Lu

**Affiliations:** 1 Department of Psychiatry, First Affiliated Hospital of Jinan University, Guangzhou, China; 2 Shenzhen Mental Health Center/Shenzhen Kangning Hospital, Shenzhen, China; 3 School of Education Science, Guangdong Polytechnic Normal University, Guangzhou, China

**Keywords:** adolescents, emotional processing, event-related potentials, longitudinal study, mismatch negativity, nonsuicidal self-injury, P3

## Abstract

**Background:**

Adolescents with nonsuicidal self-injury (NSSI) often show deficits in negative emotion regulation. Within the dual-process framework of implicit and explicit emotion regulation, these deficits may reflect an automatic bias toward negative information and insufficient later-stage controlled regulation. Whether routine clinical intervention modifies both early automatic detection and later controlled evaluation of negative emotional information remains unclear.

**Methods:**

In a longitudinal sample of 32 adolescents with NSSI, we examined changes in mismatch negativity (MMN) and P3 components elicited during an emotional oddball task before and after a 12-week clinical intervention. At baseline and Week 12, participants completed clinical assessments and a two-choice visual emotional oddball task containing neutral standards and negative, positive, and neutral deviants while EEG was recorded. MMN (Fz, 240–300 ms) and P3 (Pz, 450–650 ms) amplitudes were derived from deviant-minus-standard difference waves, indexing pre-attentive deviance detection and controlled evaluation, respectively.

**Results:**

The intervention selectively modulated neurocognitive processing of negative deviants. Self-injury ideation days (*p* = .036) and NSSI episode frequency (*p* = .007) both significantly decreased. MMN absolute amplitude to negative stimuli decreased significantly (*p* = .047), suggesting attenuated automatic salience detection, whereas P3 amplitude to negative stimuli increased significantly (p = .027), indicating enhanced controlled evaluation. Responses to positive and neutral conditions and behavioural performance remained stable. No baseline ERP–clinical correlations emerged; post-intervention, NSSI ideation days correlated with positive P3 (*r* = 0.380, *p* = .032), and episode frequency with neutral P3 (*r* = 0.461, *p* = .008).

**Conclusions:**

A 12-week intervention may attenuate automatic negative bias while enhancing controlled evaluative processing in adolescents with NSSI, consistent with the implicit–explicit framework. MMN and P3 may serve as candidate biomarkers and temporally sensitive tools for tracking intervention response when behavioural performance is stable.

## 1 Introduction

Nonsuicidal self-injury (NSSI) refers to the deliberate destruction of one’s own body tissue in the absence of suicidal intent (1) and constitutes one of the most prevalent and clinically significant mental health concerns among adolescents. In practical terms, NSSI includes behaviours such as cutting, scratching, or burning one’s skin that are performed without an intention to die, often in the context of intense emotional distress (1, 2). Lifetime prevalence estimates in community samples range from approximately 17% to 30%, with substantially greater rates reported in clinical populations (3, 4). Because NSSI commonly emerges during adolescence, when emotion-regulation and cognitive-control systems are still developing, adolescents constitute a clinically important group for mechanistic and intervention studies (5, 6). Even at subclinical levels, NSSI places individuals at increased risk for subsequent social-functional impairment, including peer relationship disruption and academic difficulties, as well as physical health consequences such as wound infection and tissue damage (1). Critically, NSSI is among the strongest proximal predictors of subsequent suicide attempts (7), emphasizing the urgency of advancing our understanding of its neurocognitive mechanisms and responsiveness to intervention. Common clinical approaches include psychotherapeutic support, such as dialectical behaviour therapy skills training, and medication management when indicated by comorbid symptoms (8, 9). However, the treatment effects of existing NSSI interventions are generally moderate, highlighting the need for further identification of potentially malleable intervention targets (9, 10). In recent years, beyond traditional psychotherapeutic and pharmacological approaches, several non-invasive physical interventions, including repetitive transcranial magnetic stimulation (rTMS) and transcranial direct current stimulation (tDCS), have been preliminarily explored as adjunctive treatments for NSSI and self-harm behaviours, with emerging evidence suggesting potential benefits for reducing self-injury urges and improving emotion regulation (41, 42). Moreover, because NSSI frequently co-occurs with depression, borderline personality disorder, and other psychiatric conditions, treatments primarily targeting these comorbid disorders, such as cognitive-behavioural therapy and mentalisation-based treatment, have likewise shown indirect efficacy in attenuating NSSI symptoms (43). Despite these expanding therapeutic options, however, the underlying neurocognitive mechanisms through which clinical interventions modify aberrant emotional processing in adolescents with NSSI remain poorly characterised.

According to cognitive models of psychopathology (11), various forms of mental disorders are expressed, at least in part, through the aberrant processing of emotional information (12, 13). In the domain of NSSI, emotion dysregulation has been consistently identified as a core pathopsychological mechanism, most prominently manifested as impaired control over negative affect. The cognitive–emotional model of NSSI (CEM–NSSI) posits that heightened emotional reactivity, coupled with impaired inhibitory control, helps explain vulnerability to self-injury as a maladaptive emotion-regulation strategy (14); a related experiential-avoidance account similarly frames self-injury as an attempt to escape or regulate aversive internal states (15). From the perspective of dual-process theories of emotion regulation, this dysregulation may simultaneously involve two relatively independent pathways: an automatic (implicit) pathway operating outside conscious awareness, whereby individuals exhibit elevated rapid detection of and automatic responsiveness to negatively salient stimuli, and a controlled (explicit) pathway involving effortful conscious evaluation and regulation, whereby individuals demonstrate deficiencies in later-stage attentional allocation, cognitive appraisal, and emotion regulation (16, 17). Recent theoretical perspectives have suggested that NSSI may involve concurrent abnormalities in both implicit and explicit emotion regulation processes (18), but direct neurophysiological evidence verifying this hypothesis remains scarce, particularly within longitudinal intervention contexts.

An emotional oddball task is a paradigm in which frequent standard stimuli establish an expected background and rare deviant stimuli test how efficiently the brain detects and evaluates unexpected emotional events. The emotional oddball paradigm provides a well-suited experimental framework for examining automatic and controlled processes during selective emotional processing. By embedding low-probability negative emotional deviant stimuli within a stream of high-probability standard stimuli, this paradigm facilitates the simultaneous assessment of pre-attentive automatic change detection and subsequent attentional allocation and evaluative processing of emotionally salient deviations. Given its millisecond-level temporal resolution, electroencephalography (EEG) is particularly well suited for capturing the dynamic temporal progression from early automatic detection to later controlled processing of negative emotional deviant stimuli. Prior event-related potential (ERP) research has demonstrated that emotional pictures can reliably modulate late positive components, while visual mismatch negativity (MMN) studies have revealed that deviant stimuli can elicit automatic change detection even in the absence of explicit attentional engagement (19–21). The emotional oddball paradigm therefore notably corresponds with the dual-process framework of emotion regulation. Within this paradigm, MMN and P3 represent two particularly informative ERP components: the former primarily reflects automatic change detection, while the latter largely reflects later-stage attentional allocation and stimulus evaluation (21, 22).

To translate this paradigm into clinically interpretable neural markers, the present study focused on MMN and P3. MMN refers to a frontocentral negative deflection peaking approximately 150–300 ms post-stimulus, which reflects pre-attentive automatic change detection that does not require active attentional engagement (23). Greater absolute amplitudes of MMN indicate stronger automatic detection of the deviation in a deviant stimulus from the standard background. MMN abnormalities have been documented across a range of psychiatric disorders, including major depressive disorder (24) and schizophrenia (25), and this component is increasingly considered a candidate biomarker for psychiatric illness. The P3 component refers to a positive deflection maximum at centroparietal electrode sites, emerging approximately 300–700 ms post-stimulus, and is associated with later-stage controlled processing, including sustained attentional allocation, stimulus evaluation, and cognitive control (22). In clinical populations characterized by emotion dysregulation, abnormalities in P3 and related late positive components have frequently been interpreted as neural signatures of impaired later-stage attentional resource allocation and evaluative processing (26–28).

Although electrophysiological research related to NSSI has increased in recent years, direct ERP evidence from NSSI populations remains limited overall, with existing studies relying primarily on the use of cross-sectional designs. Most available task-based evidence has focused either on inhibitory control in people who self-injure (29) or on emotional evaluation processes that require explicit attentional engagement in related emotion-dysregulation conditions (26, 27), whereas direct investigations of pre-attentive, automatic emotional change detection processes remain relatively rare. Thus, the specific knowledge gap addressed here is whether a routine clinical intervention can alter both implicit automatic negative deviance detection and explicit controlled evaluation in adolescents with a history of NSSI, a question that cross-sectional ERP studies cannot resolve.

The overarching aim of this study was to determine whether routine clinical intervention is associated with parallel changes in implicit automatic detection and explicit controlled evaluation of negative emotional information in adolescents with a history of NSSI. To achieve this aim, we examined changes in the amplitudes of the MMN and P3 components during an emotional oddball task before and after a 12-week clinical intervention. On the basis of the CEM-NSSI, the implicit-explicit emotion regulation framework, and existing ERP literature, we hypothesized that intervention effects would be manifested primarily under the negative emotional condition; at the pre-attentive stage, the MMN component elicited by negative stimuli would be attenuated, suggesting a reduction in automatic negative processing bias; and at the later evaluative stage, the P3 component elicited by negative stimuli would be enhanced, suggesting augmented controlled processing engagement. Given the sample size and design characteristics of this study, the results of analyses of the associations between ERP measures and clinical indicators are reported as exploratory findings. By combining longitudinal ERP assessment with an emotional oddball task, this study provides a temporally resolved test of whether intervention-related change occurs at both processing stages.

## 2 Method

### 2.1 Participants

An initial sample of 35 adolescents with a history of NSSI was recruited from the outpatient and inpatient services of Shenzhen Kangning Hospital. Participants were identified through clinician referrals and screening. All participants met the following inclusion criteria: (a) individuals were aged 12–18 years; (b) they had experienced a minimum of five NSSI episodes within the past 12 months, which is consistent with the DSM-5 research criteria for NSSI disorder (30); and (c) they possessed a sufficient cognitive capacity to complete computerized tasks and self-report measures. The exclusion criteria included the presence of (a) current psychotic symptoms, (b) intellectual disability, and (c) neurological conditions affecting the EEG recording data. Two individuals were excluded because of excessive EEG artefacts (a mean artefact epoch rejection rate exceeding 50%), and one individual was excluded because of an anomalous reaction time (exceeding five standard deviations (*SD*s) from the individual mean (*M*)). The final sample encompassed 32 individuals (30 females and 2 males; *M* = 15.75 years; *SD* = 1.57 years; range: 13–18 years). At baseline, participants reported a mean of 13.39 (SD = 1.71) days with self-injury ideation and a mean of 8.64 (SD = 2.33) NSSI episodes over the preceding four weeks. All 32 participants completed both the baseline and the Week-12 post-intervention assessments, yielding no attrition during the longitudinal follow-up. The strongly female-skewed gender distribution is consistent with the typical sex ratio observed in treatment-seeking adolescent NSSI samples but, as discussed in the Limitations (Section 4.5), restricts the external validity of the present findings. The study was approved by the Ethics Committee of Shenzhen Kangning Hospital. Written informed consent was obtained from all participants and their legal guardians. For participants under 16 years of age, parental informed consent was obtained alongside written assent from the adolescent.

### 2.2 Procedure

In this study, a longitudinal repeated-measures design was employed. During each laboratory visit, informed consent was first obtained, followed by clinical assessment and EEG recording. Participants completed the task in a dimly lit, sound-attenuated laboratory, with a research assistant applying the electrode cap and providing task instructions. The participant group was assessed at baseline (Week 0) and after 12 weeks of clinical intervention (Week 12), with each assessment comprising ERP recording and clinical evaluation processes. The intervention comprised routine multimodal clinical treatment administered according to clinical indications, encompassing pharmacotherapy and psychotherapeutic support. Each laboratory visit lasted approximately 1.5–2 hours.

### 2.3 Clinical assessment measures

At both the baseline and post-intervention, two clinical indicators directly related to NSSI were recorded: (a) the number of days with self-injury ideation over the preceding four weeks and (b) the frequency of NSSI episodes over the preceding four weeks. These indicators were used to evaluate clinical change from the pre- to postintervention stages and for subsequent correlational analyses with the amplitudes of ERP measures.

### 2.4 Emotional oddball paradigm

A two-choice visual oddball paradigm was employed. The stimuli were presented using E-Prime. The standard stimulus was a neutral object image (a black keyboard), accounting for approximately 70% of all trials (approximately 140 trials). Deviant stimuli comprised emotional scene images drawn from the Geneva Affective Picture Database (GAPED) (31), categorized into the following three types: 20 negative scenes (e.g., disaster scenes and threatening animals), 20 positive scenes (e.g., cute animals and infants), and 20 neutral scenes (e.g., natural landscapes and everyday objects). Each deviant category constituted approximately 10% of all trials (approximately 20 trials per category; approximately 60 deviant trials in total). The task sequence was adapted from previous work (32).

**Figure 1:**
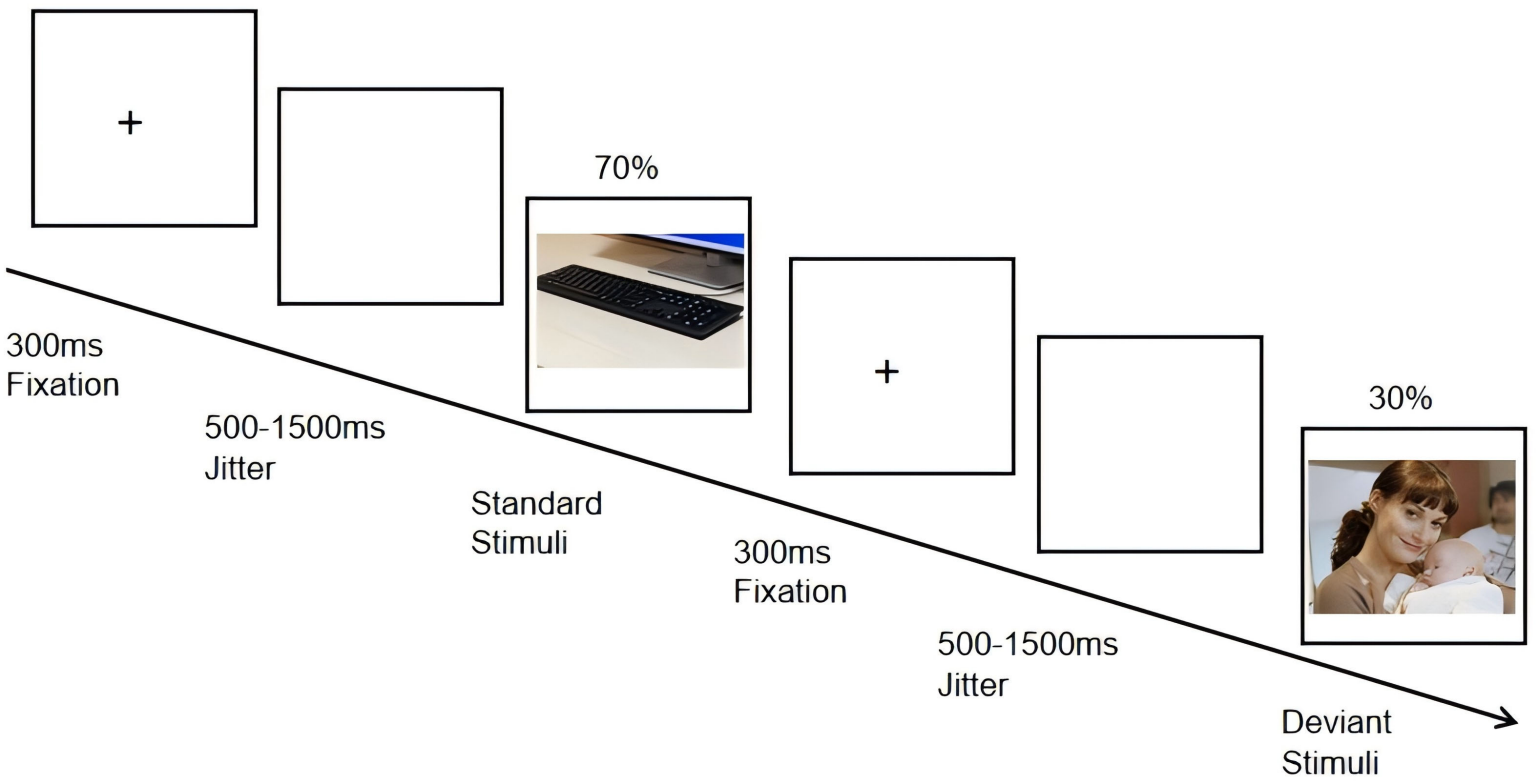
Two-choice emotional oddball task paradigm. Standard stimuli appeared in 70% of the trials, while negative, positive, and neutral emotional deviant stimuli together appeared in 30% of the trials. Source: Smiling face image retrieved from the Geneva Affective Picture Database (GAPED), used under CC BY-NC-SA 3.0 license.

Each trial began with a central fixation cross (300 ms), followed by a blank screen of randomly varying durations (500–1500 ms) to prevent temporal expectation effects, and finally the stimulus image (1000 ms). Participants were required to respond via button press action as quickly and accurately as possible during stimulus presentation or within 500 ms of stimulus offset. The task required participants to classify each stimulus as either a standard or deviant stimulus. For half of the participants, the *F* key was assigned to standard stimuli, while the J key was assigned to deviant stimuli. The remaining participants received the reverse key assignment. The 60 deviant images were randomly presented across the experimental blocks together with 140 standard trials. Each deviant image was presented only once throughout the experiment. Each image was presented only once throughout the experiment. Prior to the formal experiment, all participants completed 10 practice trials (including both standard and deviant stimuli) and were required to achieve 100% accuracy before proceeding to the formal task. The entire experiment lasted approximately 25–35 minutes. The task structure is illustrated in **Figure 1**.

### 2.5 EEG recording and preprocessing

EEG data were recorded via 32 Ag/AgCl electrodes embedded in an elastic cap (EASYCAP GmbH, Germany) positioned according to the international 10–20 system. The online reference electrode was placed at the tip of the nose, and the ground electrode was placed at the frontopolar midline (FPz). Two additional electrodes were placed at the outer canthi to monitor horizontal electrooculographic activity (HEOG), and two electrodes were positioned above and below the left eye to monitor vertical electrooculographic activity (VEOG). The sampling rate was 500 Hz, with an online bandpass filter of 0.1–100 Hz. All electrode impedances were reduced below 10 kΩ prior to recording.

Offline analysis was conducted using EEGLAB 2023 (33) on the MATLAB R2023a platform. Data were first rereferenced to the average of all scalp electrodes and then bandpass filtered at 0.1–30 Hz. Independent component analysis (ICA) was conducted to identify and remove ocular and myogenic artefact components. ICA components were selected for removal based on scalp topography, time course, and activity pattern. Continuous data were segmented in a time-locked manner to stimulus onset, with epoch window sizes ranging from -200 to 800 ms. Baseline correction was applied using the prestimulus interval (-200 to 0 ms). A semiautomatic artefact detection procedure was employed to reject any epoch in which the voltage fluctuations exceeded ±150 μV. The remaining artefacts were identified through visual inspection.

Artefact-free epochs were averaged separately for each stimulus condition. MMN and P3 were computed as difference waves (deviant minus standard). On the basis of the literature and visual inspection of the grand-average waveforms (21, 23), the following ERP components and regions of interest were defined: MMN as the mean amplitude at electrode Fz within the 240–300 ms time window and P3 as the mean amplitude at electrode Pz within the 450–650 ms time window.

### 2.6 Data analysis

MATLAB R2023a was used for all analyses with default statistical-function settings unless otherwise specified. First, paired-samples *t* tests were conducted for the number of self-injury ideation days and NSSI episode frequency to evaluate clinical change from the pre- to postintervention stages. Second, the behavioural data (accuracy and reaction time) were each subjected to a 2 (Time: baseline, post-intervention) × 4 (Conditions: negative deviant, positive deviant, neutral deviant, and standard stimuli) repeated-measures analysis of variance (ANOVA) to assess task performance across conditions. Reaction time analyses did not include error trials or trials with reaction times exceeding ±3 *SD*s from *M*. Greenhouse-Geisser corrections were applied when the sphericity assumption was violated, and Bonferroni corrections were employed for *post hoc* pairwise comparisons. Third, with respect to the ERP data, separate 2 (Time: baseline, post-intervention) × 3 (Emotion: negative, positive, and neutral emotions) repeated-measures ANOVAs were conducted for the amplitudes of MMN and P3. Finally, Pearson’s correlations between the NSSI clinical indicators (number of ideation days and episode frequency) and amplitudes of ERP measures (MMN and P3, three emotional conditions) were calculated separately at baseline and post-intervention.

## 3 Results

### 3.1 Self-injury ideation and behaviour

**Figure 2:**
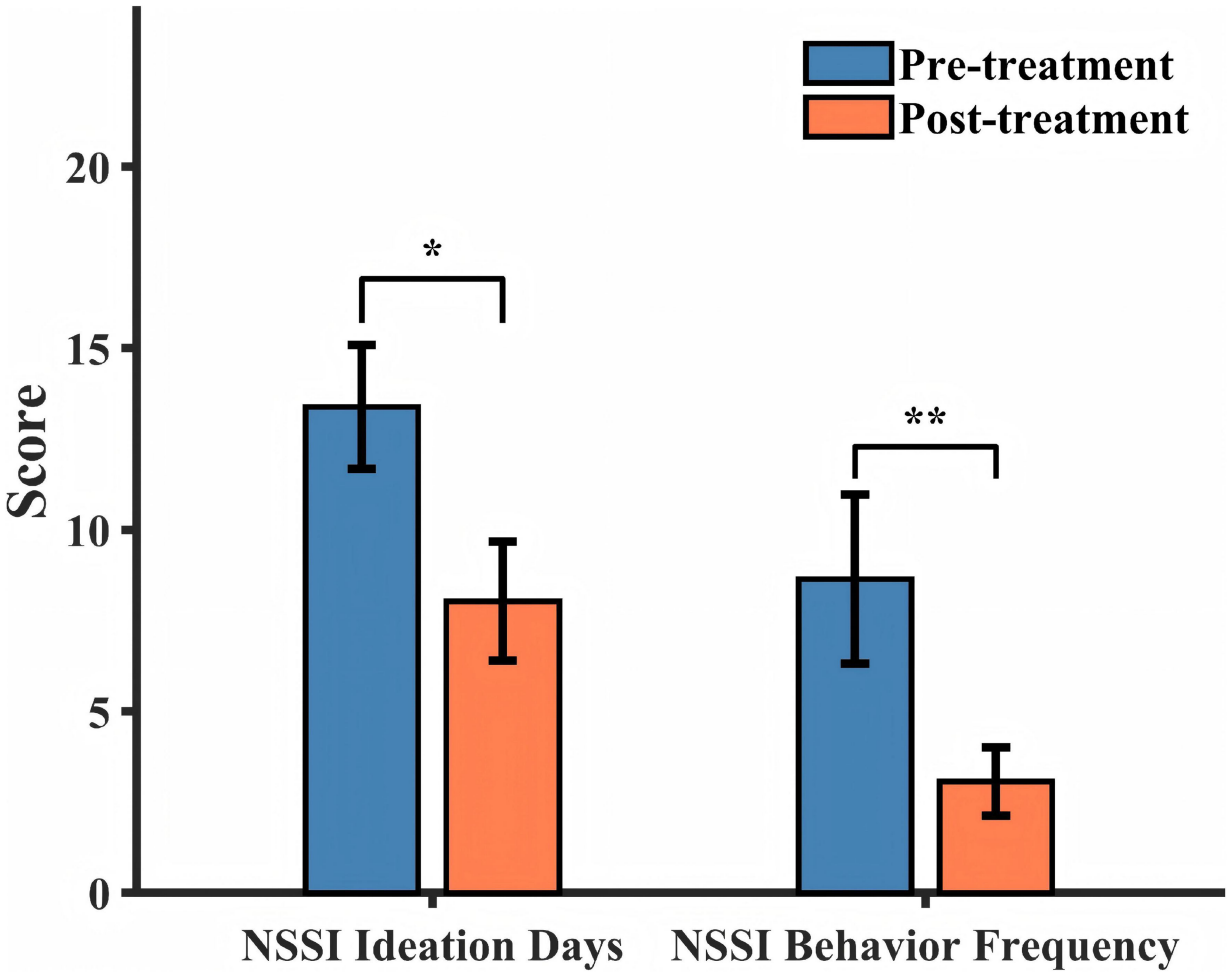
NSSI clinical indicators at baseline and postintervention. The bars indicate means, and the error bars indicate standard errors. NSSI = nonsuicidal self-injury; *p <.05, **p <.01.

The results of paired-samples tests revealed that the number of days with self-injury ideation decreased significantly from 13.39 ± 1.71 at baseline to 8.03 ± 1.64 at the postintervention stage (*p* = .036, Cohen’s *d_z_* = 0.39) and that the NSSI episode frequency decreased significantly from 8.64 ± 2.33 to 3.06 ± 0.94 (*p* = .007, Cohen’s *d_z_* = 0.51). These results indicate that treatment significantly improved both self-injury ideation and behaviour, with a more notable effect on self-injury behaviour (**Figure 2**).

### 3.2 Accuracy and reaction time

To determine whether ERP changes could be interpreted independently of task-performance changes, accuracy and reaction time data were subjected to 2 (Time) × 4 (Condition) repeated-measures ANOVAs. With respect to accuracy, the main effect of Time was not significant (*F*(1, 31) = 0.02, *p* = .896, *η_p_*^2^ = .001), indicating that overall accuracy did not change between the two time points. The main effect of Condition was significant (*F*(3, 93) = 4.24, *p* = .011, *η_p_*^2^ = .120). Bonferroni-corrected *post hoc* tests revealed that accuracy for standard stimuli was greater than that for neutral deviant stimuli (*p* = .032) and positive deviant stimuli (*p* = .021). Critically, the Time × Condition interaction was not significant (*F*(3, 93) = 0.71, *p* = .521, *η_p_*^2^ = .022).

**Figure 3:**
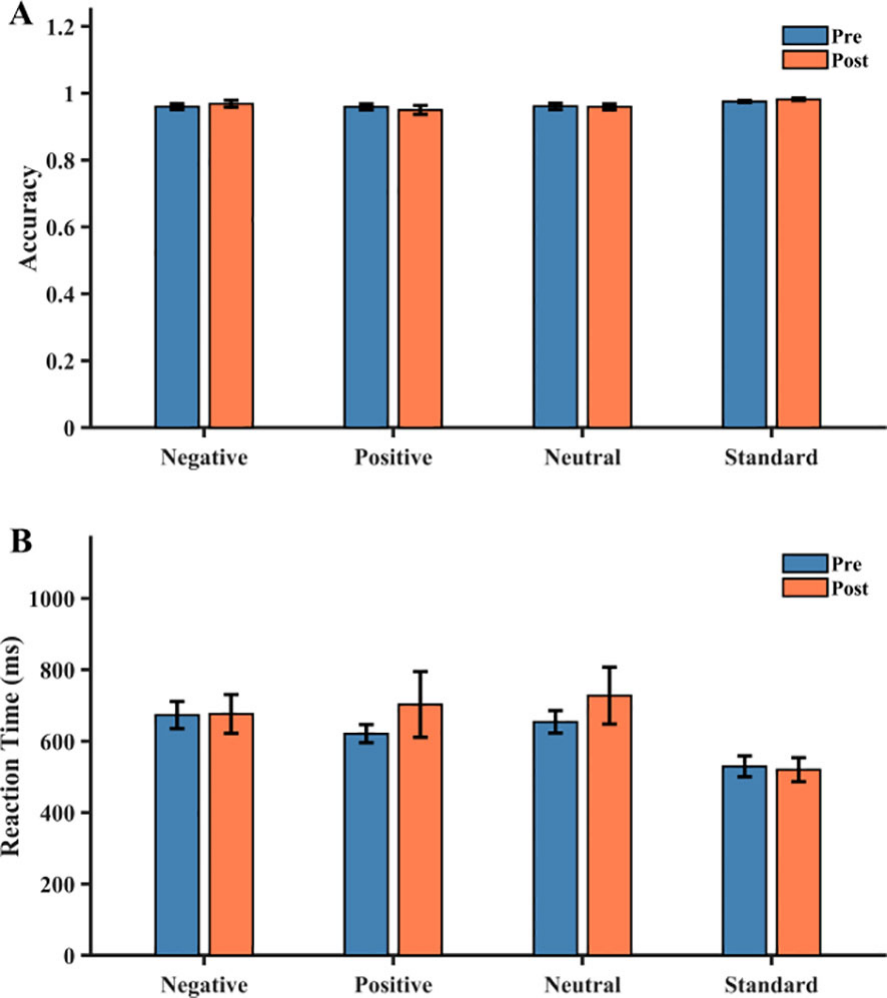
Behavioural accuracy and reaction time by condition and assessment time. **(A)** Accuracy. **(B)** Reaction time.

With respect to reaction time, the main effect of Time was not significant (*F*(1, 31) = 0.58, *p* = .451, *η_p_*^2^ = .018). The main effect of Condition was significant (*F*(3, 93) = 5.44, *p* = .005, *η_p_*^2^ = .149), with faster responses to standard stimuli than to negative deviant stimuli (*p* <.001) and neutral deviant stimuli (*p* <.001). The Time × Condition interaction was not significant (*F*(3, 93) = 0.61, *p* = .557, *η_p_*^2^ = .019) (**Figure 3**, **Table 1**). Together, accuracy and reaction time remained stable across time, indicating that the main longitudinal effects were not driven by behavioural performance changes.

**Table 1:** Accuracy and reaction time remained stable from pre- to postintervention (M ± SD).

Measure	Time	Negative	Positive	Neutral	Standard
ACC	Pre	0.96 ± 0.05	0.96 ± 0.05	0.96 ± 0.05	0.98 ± 0.02
ACC	Post	0.97 ± 0.06	0.95 ± 0.07	0.96 ± 0.05	0.98 ± 0.02
RT (ms)	Pre	673 ± 215	621 ± 144	654 ± 177	529 ± 164
RT (ms)	Post	676 ± 307	703 ± 520	728 ± 451	520 ± 189

### 3.3 ERP data

#### 3.3.1 MMN component

**Figure 4:**
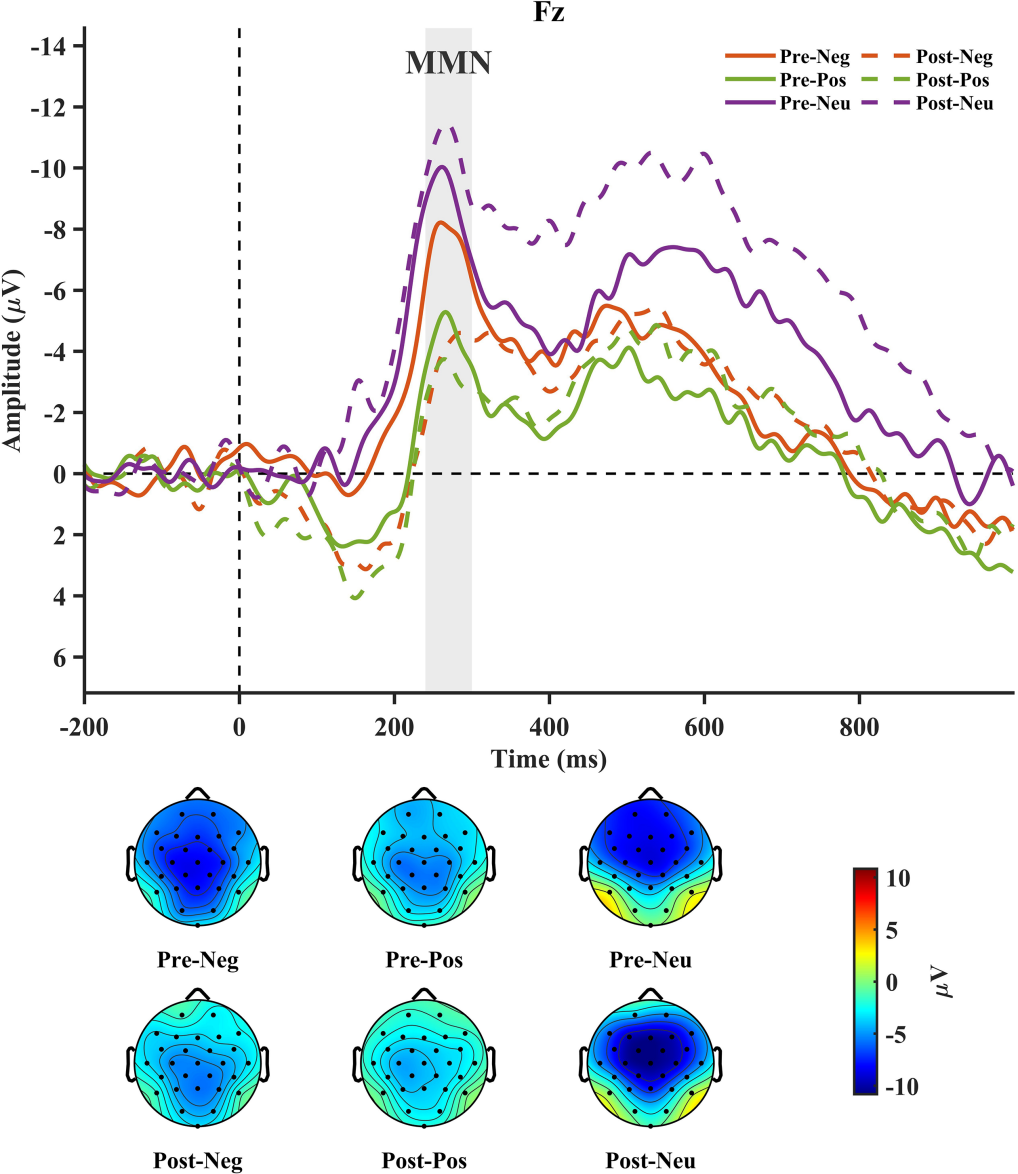
Negative-stimulus MMN difference waveforms before and after intervention. The solid lines indicate preintervention results, the dashed lines indicate postintervention results, and the shaded grey region indicates the MMN time window (240–300 ms).

To test whether the intervention changed early automatic emotional deviance detection, a 2 (Time: baseline, postintervention) × 3 (Emotion: negative, positive, and neutral emotions) repeated-measures ANOVA was conducted for the MMN amplitude (Fz electrode, 240–300 ms). The main effect of Time was not significant (*F*(1, 31) = 0.91, *p* = .347, *η_p_*^2^ = .029), indicating that there was no overall difference in MMN amplitude between the two time points. The main effect of Emotion was significant (*F*(2, 62) = 18.40, *p* <.001, *η_p_*^2^ = .372), indicating that MMN amplitude differed significantly across emotional valence conditions. Crucially, the Time × Emotion interaction was significant (*F*(2, 62) = 3.93, *p* = .029, *η_p_*^2^ = .112), indicating that the effect of the intervention on MMN amplitude varied as a function of emotional condition (**Table 2**). Simple effects analyses revealed a significant difference in MMN amplitude between the pre- and postintervention stages under the negative emotional condition (*p* = .047, Cohen’s *d_z_* = 0.37; **Table 3**; **Figure 4**). Specifically, MMN amplitude at baseline (*M* = -7.45; *SD* = 8.58) was significantly more negative than that at the postintervention stage (*M* = -3.90; *SD* = 8.06). Notably, the absolute MMN amplitude in response to negative stimuli was significantly reduced following the intervention, which suggests that treatment attenuated the automatic detection response of the brain to negative deviant stimuli. MMN amplitudes under positive (*p* = .604) and neutral (*p* = .406) emotional conditions did not differ significantly between the two time points. Thus, the MMN findings show an emotion-specific reduction in automatic detection of negative deviant stimuli after intervention.

**Table 2:** Time × Emotion interactions showed emotion-specific intervention effects for MMN and P3 amplitudes.

Component	Effect	*df*	*F*	*p*	*η_p_* ^2^
MMN (Fz)	Time	1, 31	0.91	.347	.029
MMN (Fz)	Emotion	2, 62	18.40	<.001***	.372
MMN (Fz)	Time × Emotion	2, 62	3.93	.029*	.112
P3 (Pz)	Time	1, 31	0.37	.550	.012
P3 (Pz)	Emotion	2, 62	3.51	.038*	.102
P3 (Pz)	Time × Emotion	2, 62	4.40	.017*	.124

**Table 3:** Negative MMN decreased and negative P3 increased after intervention across emotional conditions (M ± SD, μV).

Component	Emotional condition	Pre-intervention	Post-intervention	*p*
MMN (Fz)	Negative	−7.45 ± 8.58	−3.90 ± 8.06	.047*
MMN (Fz)	Positive	−4.18 ± 6.65	−3.50 ± 8.10	.604
MMN (Fz)	Neutral	−9.13 ± 5.65	−10.25 ± 5.42	.406
P3 (Pz)	Negative	1.01 ± 5.99	3.64 ± 6.60	.027*
P3 (Pz)	Positive	4.15 ± 6.70	5.20 ± 5.02	.404
P3 (Pz)	Neutral	5.23 ± 7.99	3.23 ± 5.32	.193

#### 3.3.2 P3 component

**Figure 5:**
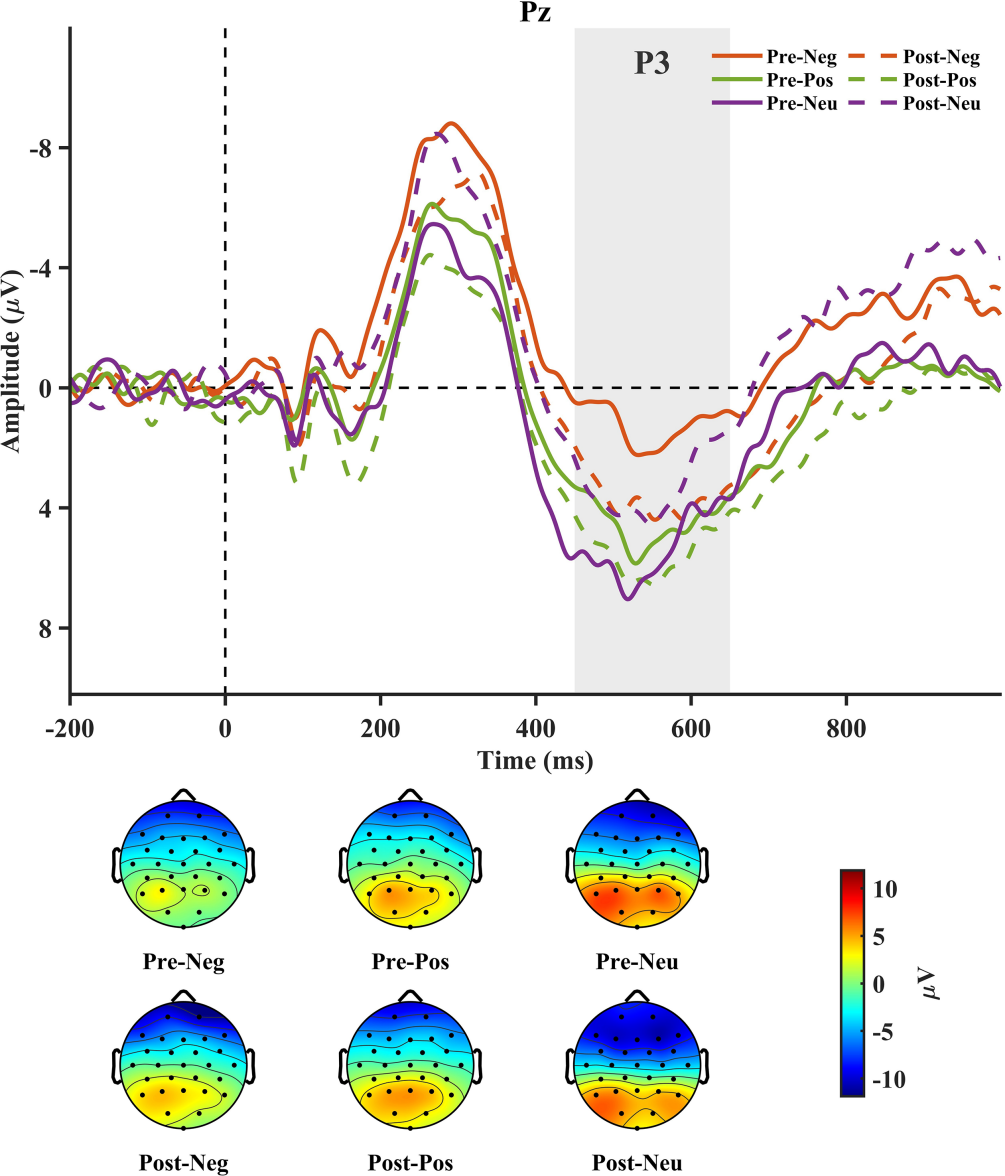
Negative-stimulus P3 difference waveforms before and after intervention. The solid lines indicate preintervention results, the dashed lines indicate postintervention results, and the shaded grey region indicates the P3 time window (450–650 ms).

To test later-stage controlled evaluative processing, an identical 2 × 3 repeated-measures ANOVA was conducted for the P3 amplitude (Pz electrode, 450–650 ms). The main effect of Time was not significant (*F*(1, 31) = 0.37, *p* = .550, *η_p_*^2^ = .012). The main effect of Emotion was significant (*F*(2, 62) = 3.51, *p* = .038, *η_p_*^2^ = .102). The Time × Emotion interaction was significant (*F*(2, 62) = 4.40, *p* = .017, *η_p_*^2^ = .124), indicating that the effect of the intervention on P3 amplitude was emotion specific (**Table 2**). Simple effects analyses revealed a significant difference in P3 amplitude between the pre- and postintervention stages under the negative emotional condition (*p* = .027, Cohen’s *d_z_* = 0.41; **Table 3**; **Figure 5**). Specifically, the postintervention P3 amplitude (*M* = 3.64; *SD* = 6.60) was significantly greater than the preintervention amplitude (*M* = 1.01; *SD* = 5.99), suggesting that the intervention enhanced controlled evaluative processing of negative emotional stimuli. P3 amplitudes under positive (*p* = .404) and neutral (*p* = .193) emotional conditions did not differ significantly between the two time points. These data indicate that P3 captured treatment-related change specifically in the negative emotional condition, rather than reflecting a generalized change across emotional categories.

### 3.4 Correlations between self-injury indicators and ERP amplitudes

**Figure 6:**
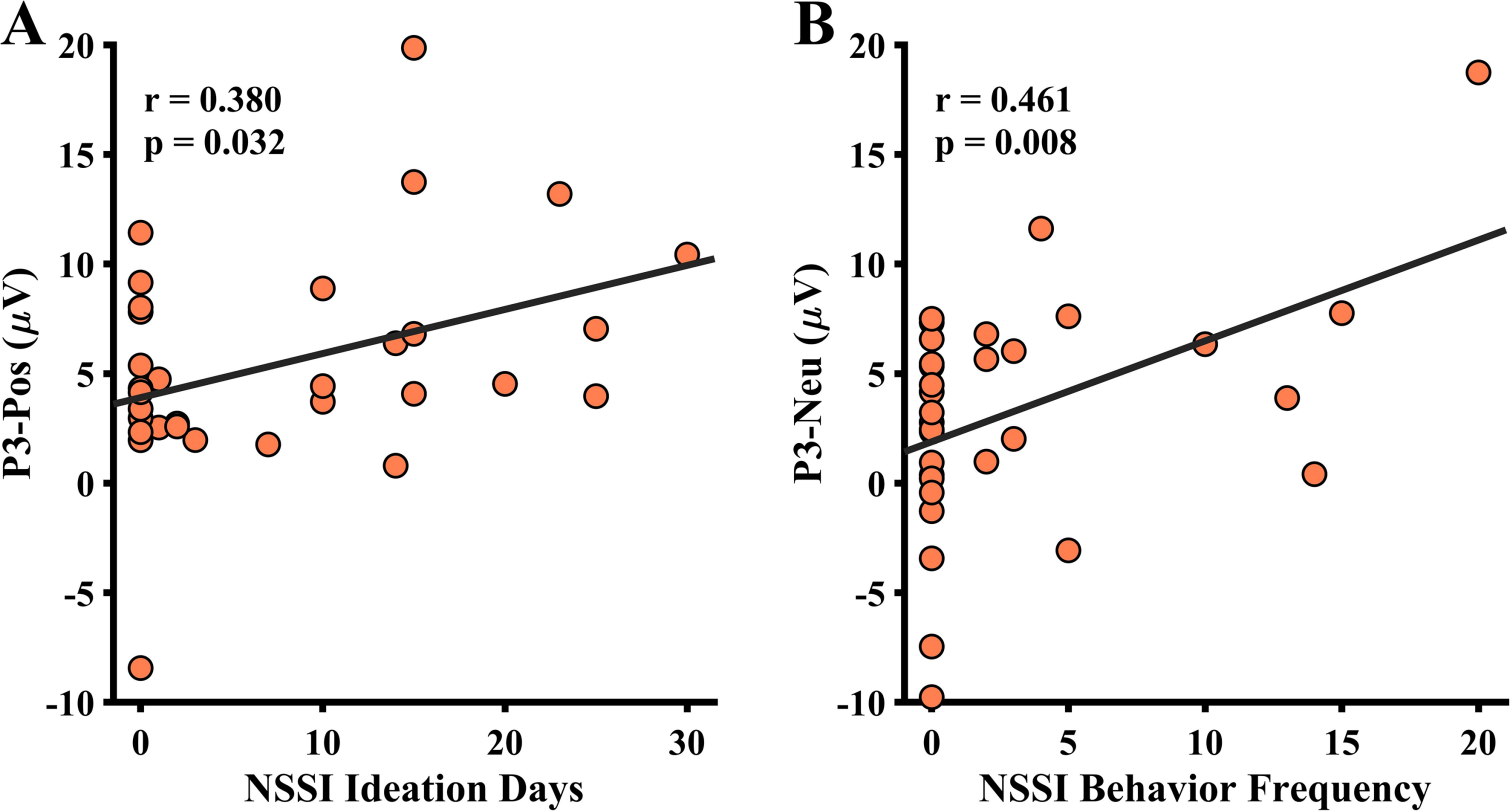
Postintervention P3-clinical indicator scatterplots. **(A)** shows the correlation between the number of NSSI ideation days and the positive P3 amplitude, and **(B)** shows the correlation between NSSI episode frequency and the neutral P3 amplitude.

To explore whether neural indices were related to symptom-level change, Pearson correlations were calculated separately at baseline and postintervention. At baseline, no statistically significant correlations were obtained between clinical indicators and ERP amplitudes (all *p* values >.05). At the postintervention stage, two significant correlations emerged: the number of NSSI ideation days was significantly correlated with the positive P3 amplitude (*r* = 0.380, *p* = .032), and NSSI episode frequency was significantly correlated with the neutral P3 amplitude (*r* = 0.461, *p* = .008) (**Figure 6**). Overall, significant correlations were observed only at the postintervention stage rather than at baseline, suggesting that the process of clinical improvement may facilitate emergent coupling between neural and symptom-level indices. Together, the correlation findings should be interpreted as exploratory evidence that residual symptoms may be linked to later-stage evaluative processing after intervention.

## 4 Discussion

Consistent with the overarching aim of determining whether routine clinical intervention is associated with parallel changes in implicit automatic detection and explicit controlled evaluation of negative emotional information, this study used longitudinal ERP data obtained before and after a 12-week clinical intervention in adolescents with a history of NSSI who completed an emotional oddball task. The results revealed that intervention effects were concentrated primarily on negative emotional deviant stimuli. Notably, the negative MMN amplitude decreased significantly, and the negative P3 amplitude increased significantly. In contrast, ERP amplitudes under positive and neutral emotional conditions did not change significantly, and behavioural accuracy and reaction time likewise remained stable. Furthermore, no significant correlations between clinical indicators and ERP amplitudes were observed at baseline, whereas significant associations between P3 amplitude and NSSI clinical indicators emerged postintervention. Overall, these results suggest that changes that occurred in adolescents with a history of NSSI over the 12-week follow-up period were manifested primarily at the neural level of negative emotional processing, characterized by the co-occurrence of attenuated automatic detection bias and enhanced controlled evaluative processing.

### 4.1 Dual-process framework of emotion regulation in individuals who engage in NSSI

The emotion-specific MMN-P3 pattern supports a dual-process interpretation of NSSI-related emotion dysregulation. NSSI is widely regarded as a clinical phenomenon closely tied to emotion dysregulation, with the most salient feature being impaired control over negative affect (14, 18, 32). The CEM-NSSI posits that elevated emotional reactivity, coupled with compromised inhibitory control, constitutes a key vulnerability pathway for NSSI (14); related experiential-avoidance accounts further emphasize self-injury as an attempt to escape aversive internal states (15). According to the implicit-explicit emotion regulation framework, this dysfunction may manifest as distinct patterns of abnormality at different processing stages: at earlier stages, individuals may exhibit a heightened automatic detection bias towards negative emotional cues such that negative information more readily surpasses the sensory threshold and gains preferential access to processing resources; at later stages, individuals may lack sufficient effective conscious appraisal and regulatory capacity, resulting in difficulty when reappraising or inhibiting emotional responses is needed (16, 17, 34). By linking these stages to treatment-associated change, the present findings extend dual-process accounts from cross-sectional vulnerability models to a longitudinal clinical context.

In this study, the emotional oddball paradigm was employed in conjunction with MMN and P3 indices to separate these two levels along the temporal processing continuum. By embedding low-probability negative emotional deviant stimuli within a stream of high-probability standard stimuli, the emotional oddball paradigm activates both pre-attentive automatic change detection and subsequent controlled evaluative processing within a single experimental framework. As a pre-attentive component, MMN reflects sensory-memory-based deviance detection that does not require active attentional engagement. In contrast, P3, as a later component, reflects conscious attentional allocation, stimulus evaluation, and associated cognitive control processes. This temporal-component dissociation maps well onto the implicit–explicit dual-process framework (21, 22).

The central finding of this study is that postintervention changes did not emerge uniformly across all emotional conditions but were instead concentrated on the MMN and P3 components elicited by negative deviant stimuli. The ERP amplitudes under positive and neutral emotional conditions did not significantly change from the pre- to postintervention stages. This emotional specificity suggests that the neural changes observed following the 12-week clinical intervention more likely reflect a selective adjustment in the processing of negative emotional information, rather than a generalized enhancement or attenuation of emotional reactivity. From this perspective, the core difficulty for adolescents who engage in NSSI may not reside in the aberrant processing of all emotional stimuli per se but rather in a characteristic processing pattern when confronted with negative cues, i.e., one characterized by the co-occurrence of excessive automatic bias and insufficient later-stage regulatory engagement.

### 4.2 Attenuation of automatic negative processing bias after the 12-week follow-up

Consistent with the primary aim of evaluating implicit automatic processing, the negative MMN result provides evidence for attenuation of automatic negative salience detection. The absolute amplitude of MMN elicited by negative deviant stimuli decreased significantly following the intervention, whereas MMN amplitudes under positive and neutral emotional conditions did not change significantly. MMN is generally considered a pre-attentive index of automatic detection, reflecting the automatic registration of discrepancies between deviant stimuli and the standard background at the level of sensory memory (21, 23). Greater absolute amplitudes of MMN typically indicate that the brain has assigned greater automatic salience weighting to deviant information. Therefore, the reduction in the absolute amplitude of MMN under the negative emotional condition suggests that, following the intervention, the early automatic detection response of participants to negative emotional cues was attenuated, i.e., the automatic salience weighting assigned to negative information may have decreased. This finding adds longitudinal ERP evidence that automatic negative processing bias may be a modifiable neural feature in adolescents with a history of NSSI.

In light of the theoretical perspectives that position elevated negative emotional reactivity and maladaptive emotion regulation as central to NSSI (14, 15), these findings carry theoretical significance. At baseline, the participants exhibited a relatively large absolute amplitude of MMN in response to negative deviant stimuli, suggesting that their sensory processing system may have possessed an increased automatic capture tendency for negative cues. This mechanism can be understood as a form of negativity bias at the implicit emotion regulation level, i.e., even in contexts not requiring explicit attentional engagement, negative information was more likely to break through the standard background and gain preferential processing. Following the 12-week follow-up period, this automatic capture tendency was attenuated, and the participants no longer exhibited the same intensity of pre-attentive detection response to negative stimuli as observed at baseline. These findings resonate with prior work in the domains of depression and anxiety, in which treatment-related attenuation of implicit attentional bias has been reported as an important indicator of emotional disorder recovery (35, 36).

Notably, this effect was observed only at the ERP level but not in the behavioural data. As shown in **Table 1**, classification accuracy was uniformly high across all conditions (approximately 95%–98%) at both time points; this pattern is best characterised as a ceiling effect, which left essentially no measurable headroom for detecting longitudinal change and directly explains why the behavioural measures were less sensitive to pre-to-post change than the ERP measures. The two-choice classification task employed in this study required participants to distinguish between standard and deviant emotional stimuli, with the task difficulty being relatively low and the accuracy rates approaching ceiling levels across all conditions. Under these circumstances, behavioural measures provided virtually no room for detectable change, rendering them insensitive to relatively subtle processing-level adjustments from the pre- to postintervention stages. In contrast, the temporal resolution of the ERP data with respect to processing stages far exceeds that of behavioural indices, facilitating continued identification of potential changes in neural activity even when the response accuracy has already reached saturation. The clinical utility of ERP measures for detecting neural indices of symptom-relevant processing has been emphasized in clinical psychophysiology (37), suggesting that ERP measures may possess greater sensitivity for capturing subclinical changes early in the treatment process. The present MMN findings therefore strongly support an interpretation of neural-level change, specifically the attenuation of automatic negative processing bias, whereas the absence of corresponding behavioural change can more likely be attributed to the inherent sensitivity constraints of the task design itself.

### 4.3 Enhanced later-stage cognitive processing as reflected by P3 augmentation

Complementing the decrease in the absolute amplitude of MMN under the negative emotional condition, the P3 amplitude under the negative emotional condition increased significantly following the intervention, whereas P3 amplitudes under the positive and neutral emotional conditions did not change significantly. These results suggest that the clinical intervention not only influenced early automatic detection of negative emotions but also modulated later-stage controlled processing and that such later-stage processing changes were similarly emotion specific.

The P3 component is widely associated with later-stage attentional resource allocation, stimulus evaluation, and cognitive control processing (22). Within the oddball paradigm, the amplitude of the P3 component is typically proportional to the attentional resources allocated to the deviant stimulus: larger P3 amplitudes reflect greater processing capacity devoted by the individual to the deviant stimulus, thereby incorporating it into the processes of conscious evaluation and categorization (38, 39). In emotional processing research, P3 modulation is typically considered to reflect the degree of sustained attention to emotional stimuli, meaning evaluation, and regulation-related cognitive engagement (19, 26, 27).

In terms of the clinical characteristics of NSSI, the P3 amplitude under the negative emotional condition at baseline was notably smaller than that observed concurrently under the positive and neutral emotional conditions. This pattern is consistent with prior observations of emotion dysregulation populations, namely, when confronted with negative stimuli, individuals with emotion dysregulation generally exhibit relatively insufficient mobilization of later-stage attentional and evaluative resources rather than a straightforward overreaction. This result may reflect a characteristic processing deficit, namely, having already mounted a strong automatic response to negative information, the individual fails to effectively mobilize cognitive resources at subsequent stages for reappraisal or regulation, thereby leaving the emotional experience confined to the negative state initially triggered by automatic activation, without effective management through conscious processing pathways (27, 28). The significant increase in the negative P3 amplitude following the intervention thus suggested that this state of later-stage processing insufficiency was ameliorated: participants began to deploy greater attentional and evaluative resources when confronted with negative emotional cues, thereby shifting the processing trajectory from passive automatic activation towards more active cognitive engagement.

Integrating the changes in MMN and P3 within a unified temporal processing framework reveals a theoretically coherent pattern of change. At baseline, the processing profile of individuals with a history of NSSI was characterized by increased early automatic detection alongside attenuated later-stage controlled processing. Following the intervention, both stages revealed changes consistent with a recovery trajectory, namely, early automatic detection was attenuated, and later-stage controlled processing was augmented. This bidirectional modulation pattern has important theoretical implications: when only a decrease in MMN was observed, it remains unclear whether such a change represents mere response attenuation or more adaptive processing reorganization. However, the concurrent increase in P3 provides complementary evidence indicating that, following the intervention, the participants were not simply responding less to negative information or exhibiting attentional disengagement. Instead, they were more likely to shift the locus of processing from early automatic capture towards later conscious evaluation and elaboration. Within the dual-process emotion regulation framework, this result suggests that the attenuation of negativity bias at the implicit level and the augmentation of regulatory processing at the explicit level occurred in parallel, jointly indicating a more balanced mode of emotional information processing.

Furthermore, the finding that P3 changes emerged exclusively under the negative emotional condition and not under positive or neutral emotional conditions merits emphasis. If P3 amplitudes had increased across all conditions following the intervention, the change would more likely reflect a diffuse, nonspecific mobilization of general attentional resources or an increase in tonic arousal. However, the emotional selectivity of P3 in this study indicates that the later-stage processing changes facilitated by the intervention did not constitute a generalized effect but rather a specific adjustment centred on the core clinical difficulty of NSSI, i.e., the processing and regulation of negative emotion. These findings further support the theoretical premise of the CEM-NSSI in terms of the central role of negative emotional processing (14) and provide a processing-level indicator with emotional specificity for future intervention research.

Given the single-arm design, it is reasonable to ask whether the observed ERP changes simply reflect non-specific habituation or practice effects from repeated task exposure. Several features of the present pattern argue against this account. Generic habituation would predict a broad attenuation of neural responses across conditions, whereas the post-intervention changes were strictly confined to the negative emotional condition. More importantly, the negative-stimulus P3 amplitude increased rather than decreased; a simple habituation or practice account predicts an overall reduction of the late positive response with repeated testing, so the observed direction of change is opposite to that prediction and instead aligns with enhanced controlled evaluative engagement for negative emotional content. Together, the condition-specific and bidirectional MMN–P3 pattern is more consistent with a meaningful reorganisation of emotional processing than with passive repetition-driven attenuation. Nevertheless, the absence of a control group precludes strong causal claims, and the present interpretation should be regarded as converging evidence rather than as a definitive demonstration of intervention causality.

### 4.4 Emergence of ERP–clinical indicator associations after the intervention

At baseline, no correlations between clinical indicators and ERP amplitudes reached statistical significance. Following the intervention, two significant associations emerged: the number of NSSI ideation days was positively correlated with P3 amplitude under the positive emotional condition, and NSSI episode frequency was positively correlated with P3 amplitude under the neutral emotional condition.

The observation that correlational relationships emerged only at the postintervention stage rather than at baseline suggests that the treatment process may have caused interindividual differentiation. At baseline, group-level symptom indices were relatively high, and individual variations in clinical presentation and neural processing may have resulted in insufficient systematic correspondence between the two classes of indices. After 12 weeks of intervention, symptom indices decreased, and participants may have diverged in terms of the degree of symptom improvement and the pace of neural processing recovery, thereby allowing covariation between the clinical and ERP indices to emerge. This interpretation remains exploratory, but it suggests that postintervention associations between ERP and clinical indices may become identifiable only after intervention-induced expansion of individual differences allows their detection.

Directly, the present data showed that both significant postintervention correlations involved P3 rather than MMN and involved positive and neutral emotional conditions rather than the negative emotional condition. The following interpretation is therefore inferential: residual symptoms after intervention may not simply reflect persistent aberrant negative emotional processing, but may also relate to how efficiently individuals engage in later-stage evaluative processing of nonnegative information. Prior research indicates that emotion dysregulation and self-injury are associated not only with difficulty coping with negative affect but also with broader disturbances in affective and reward-related processing (18, 40). In this study, participants with more severe residual symptoms postintervention exhibited greater P3 amplitudes under both positive and neutral emotional conditions, suggesting that they may have needed to mobilize greater later-stage cognitive resources to process relatively low-threat stimuli. With respect to positive emotional stimuli, the elevated P3 may reflect additional cognitive effort during meaning evaluation of positive information. With respect to neutral emotional stimuli, a larger P3 may indicate persistent difficulty in classifying or filtering routine environmental cues, thereby requiring greater attentional resources for low-salience stimuli.

The core effects of the intervention acted primarily on the negative emotional channel (as indicated by changes in MMN and P3 under the negative emotional condition), whereas the severity of residual symptoms was more closely associated with the efficiency of later-stage processing of nonnegative information. This dissociation has clinical implications: it suggests that even when automatic negative bias and controlled processing of negative information have experienced positive adjustment during treatment, some participants may continue to exhibit relatively inefficient processing of positive information at the evaluative level and of neutral information at the routine processing level and that the latter may be related to symptom persistence or relapse risk.

### 4.5 Limitations and future directions

Several limitations of this study should be acknowledged. First, the absence of an untreated or healthy comparison group precludes definitive causal attribution of pre-to-post differences to clinical intervention per se and also limits the generalisability of the findings, which should therefore be regarded as preliminary. Second, the routine multimodal intervention was ecologically valid but varied across participants in type and intensity, limiting inferences about specific treatment components. Third, the sample was modest and predominantly female (30 females, 2 males); although broadly consistent with the gender ratio of treatment-seeking adolescent NSSI samples, this distribution restricts external validity, and the findings may not generalise to male adolescents or to gender-balanced clinical samples. Fourth, the two primary clinical indicators—self-injury ideation days and NSSI episode frequency over the preceding four weeks—were self-reported with a four-week recall window and may have been subject to recall bias; future studies should complement them with more objective or ecological-momentary measures.

## 5 Conclusions

From a longitudinal perspective, the present findings tentatively suggest that a 12-week clinical intervention was associated with selective changes in the neural processing of negative emotional deviants in adolescents with a history of NSSI. Specifically, the reduced absolute amplitude of the negative MMN may indicate attenuation of the automatic negative processing bias, whereas the enhanced negative P3 amplitude may reflect strengthened later-stage controlled evaluation. This dual pattern aligns with the implicit–explicit emotion regulation framework and supports the view that NSSI-related deficits in negative emotion regulation may operate at both automatic and controlled processing stages. The convergent modulation of MMN and P3 further reinforces the notion that emotion-processing markers may bridge symptom-level improvement and underlying neurocognitive mechanisms in adolescent NSSI. Overall, MMN and P3 show promise as candidate biomarkers for characterizing emotional processing dynamics in NSSI and for evaluating intervention efficacy. Given the single-arm design, the modest and predominantly female sample, and the absence of a healthy comparison group, however, these conclusions should be interpreted as preliminary and hypothesis-generating rather than as definitive demonstrations of treatment-specific effects. Future controlled studies with larger samples are warranted to verify these effects and to examine whether MMN and P3 indices can predict individualized treatment response and long-term clinical outcomes.

## Data Availability

The raw data supporting the conclusions of this article will be made available by the authors, without undue reservation.
